# CCR5/CCR6 Chemokine Receptor Profiles Differ in γδTCR Intensity-Defined Subsets but Remain Conserved Across Conventional and NK-like γδT Cells During Healthy First-Trimester Pregnancy

**DOI:** 10.3390/biology15161386

**Published:** 2026-08-13

**Authors:** Banan Alrawashdeh, Pál Jáksó, Barbara Forró, Ákos Várnagy, Alíz Barakonyi

**Affiliations:** 1 Department of Medical Microbiology, University of Pécs Medical School, Clinical Center, H-7624 Pécs, Hungary; alrawashdeh.banan@edu.pte.hu; 2 Department of Pathology, University of Pécs Medical School, Clinical Center, H-7624 Pécs, Hungary; jakso.pal@pte.hu (P.J.); forro.barbara@pte.hu (B.F.); 3 Department of Obstetrics and Gynaecology, University of Pécs Medical School, Clinical Center, H-7624 Pécs, Hungary; varnagy.akos@pte.hu; 4 National Laboratory on Human Reproduction, University of Pécs, H-7622 Pécs, Hungary

**Keywords:** first-trimester pregnancy, peripheral blood, γδT cells, NK-like γδT cells, CD4/CD8 phenotype, CCR5/CCR6 co-expression, chemokine receptors

## Abstract

During pregnancy, immune tolerance toward the semi-allogeneic fetus must be established. γδT cells constitute a distinct T-cell lineage characterized by expression of the γδT-cell receptor (γδTCR). They combine rapid innate-like responses with adaptive immune functions and contribute to feto-maternal immune regulation; however, their peripheral phenotypic characteristics, including chemokine receptor (ChR) profiles potentially relevant to decidual recruitment, remain incompletely characterized. Peripheral blood from healthy non-pregnant and first-trimester pregnant women was analyzed by flow cytometry to assess γδT-cell phenotypes and CCR5/CCR6 ChR expression. Analysis of γδT-cell subsets defined by lower (γδTCR^dim^) or higher (γδTCR^bright^) surface γδTCR expression showed that CCR5^−^/CCR6^−^ cells were more frequent in γδTCR^bright^ cells, whereas CCR5^+^ phenotypes were more prominent in γδTCR^dim^ cells; CD4/CD8 and CD56/CD8 distributions were comparable. Resting γδT cells were mainly CD4^−^/CD8^−^, whereas recently activated CD69^+^ γδT cells had increased CD4^+^ or CD8^+^ single-positive frequencies. Compared with non-γδT cells, γδT cells showed distinct CD4/CD8-linked CCR5/CCR6 organization, including a CCR5^+^/CCR6^−^ preference. Unlike non-γδT cells, conventional CD56^−^ and NK-like CD56^+^ γδT cells retained similar CCR5/CCR6 profiles across CD4/CD8-defined subsets. PMA/ionomycin-stimulated samples generally exhibited lower CCR5^+^ and higher CCR5^−^ frequencies across CD4^−^ conventional and NK-like γδT cell subsets. Pregnancy-related circulating γδT-cell phenotype changes were negligible, possibly reflecting decidual immune adaptation. Overall, the study highlights γδTCR intensity-linked ChR heterogeneity and, in contrast to non-γδT cells, conserved CCR5/CCR6 organization across CD56-defined γδT-cell subsets. Broader ChR phenotyping and functional migration analyses could clarify the biological relevance of these subset-specific differences.

## Simple Summary

Pregnancy is an immunological challenge because the mother’s immune system must adapt to the developing embryo, which carries genetic material from the father. Maternal immune cells increase in number in the placenta, where they help support pregnancy; however, it is still unclear where these cells originate. The embryo can produce soluble molecules that guide immune cells by binding to migration-related surface structures called chemokine receptors (ChRs); therefore, studying ChR patterns on peripheral immune cells may help us understand how these cells move. γδT cells are rare in the blood but become enriched in tissues, including the placenta, where they may contribute to fetal growth. We studied various circulating γδT cell subgroups and found that some had distinct ChR profiles, suggesting different migratory potential. This may mean that certain γδT cell types are more likely than others to move into tissues and perform relevant functions there. We also found that γδT cells differed from other T cells, highlighting their unique features. Interestingly, these γδT cell characteristics remained unchanged during the first trimester of pregnancy, suggesting that the local placental environment may help retain immune cells at the maternal–fetal interface.

## 1. Introduction

The presence of a genetically distinct embryo that expresses paternal foreign antigens within the maternal uterus has long been considered an immunological paradox. It is now well established that the maternal immune system undergoes dynamic adaptations that actively facilitate embryo implantation while maintaining host defense [1]. γδT cells form an unconventional T cell subset that plays an active role in establishing and maintaining maternal–fetal immune tolerance during pregnancy [2]. They represent a distinct T-cell lineage defined by the expression of a γδ rather than an αβT-cell receptor (TCR). Their lineage commitment occurs during early thymic development, when TCRγ, TCRδ and TCRβ gene rearrangements occur in parallel and successful receptor expression together with TCR signal strength determines γδ versus αβ lineage commitment. Unlike αβT cells, γδT cells recognize stress-associated and non-peptide ligands largely independently of classical peptide-MHC-restricted antigen presentation. They preferentially localize to epithelial and mucosal tissues, where they mediate rapid cytotoxic and cytokine responses and, depending on the subset and tissue microenvironment, contribute to tissue homeostasis and immune regulation by promoting tissue repair and, in a context-dependent manner, limiting excessive immune responses [3]. γδT cells constitute only ~1–10% of peripheral blood lymphocytes in human adults; however, their proportions are substantially higher within mucosal and epithelial tissues [4]. The expression of CD4/CD8 co-receptors is widely used to characterize lineage and functional specialization in conventional αβT cells [5]. Human peripheral blood γδT cells predominantly display a CD4^−^/CD8^−^ phenotype, while smaller subsets express only CD8 and, less commonly, CD4 [6]. Based on the intensity of γδTCR surface expression, γδT cells can be distinguished into γδTCR^dim^ and γδTCR^bright^ subsets; however, whether this distinction reflects differences in other phenotypic characteristics or functional properties remains unclear. In addition, they share several receptors with NK cells, including CD56, a marker often linked to cytotoxic activity [7]. The phenotypic and functional heterogeneity places γδT cells at the boundary between innate and adaptive immunity. Data on CD4- or CD8-expressing peripheral blood γδT cells remain limited during pregnancy; however, we previously examined the expression patterns of CD4 and CD8 co-receptors within γδT cell subsets across trimesters. The study demonstrated that CD56^+^ NK-like and CD56^−^ conventional γδT cells display distinct CD4/CD8 co-receptor profiles throughout pregnancy, with the NK-like subset typically showing higher proportions of CD4^−^/CD8^−^ and CD8 single-positive cells, whereas conventional γδT cells showed a greater relative representation of the CD4 single-positive phenotype [8].

During early human pregnancy, immune cells accumulate in the decidua, where NK cells constitute the predominant population [9]. γδT cells are enriched in the decidua, suggesting a tissue-associated phenotype [10,11]. They may contribute to maternal–fetal immune tolerance through the production of IL-10 and TGF-β, the expression of inhibitory and regulatory molecules, and their interactions with trophoblasts and decidual stromal cells [2]. However, the origin of decidual γδT cells remains incompletely understood.

Chemokine and chemokine receptor (ChR) interactions play pivotal roles in coordinating intercellular crosstalk and leukocyte migration during pregnancy, contributing to tissue development and maintenance of the decidual microenvironment [12]. Several studies have demonstrated that chemokines and ChRs are highly expressed at the fetomaternal interface by trophoblast cells, decidual stromal cells, and resident immune cells, thereby establishing a complex immune network [13]. These molecules participate in the regulation of trophoblast invasion, decidualization, and immune cell recruitment [14]. Current evidence indicates that the expression of individual ChRs is differentially regulated throughout the menstrual cycle, suggesting a regulatory role for ovarian steroid hormones, including progesterone and estrogen [15]. In this context, C-C motif chemokine receptor 5 (CCR5) mRNA levels are markedly upregulated during the luteal phase, with a 612-fold increase peaking in the premenstrual endometrium [15]. C-C motif chemokine ligand 5 (CCL5) expression in human decidual stromal cells, unlike in murine models, is not reduced during decidualization [16]. These observations suggest that the role of the CCR5–CCL5 axis in regulating T cell recruitment during human pregnancy remains to be fully elucidated. Furthermore, CCR6 is a widely used marker to distinguish γδT17 cells, which are major innate-like sources of IL-17A, from IFN-γ-producing γδT cells [17]. Studies in porcine models showed that signaling through the ChR CCR6 and its chemokine ligand CCL20 promotes the proliferation of endometrial epithelial cells, potentially preparing the endometrium for placentation and implantation [18]. In a murine model of pregnancy, elevated decidual CCL20 levels, together with increased CCR6 expression on thymic and peripheral regulatory T (Treg) cells, suggest that the CCL20–CCR6 axis contributes to the chemokine-mediated recruitment of Treg cells to the fetomaternal interface [19]. Additionally, altered plasma levels of CCL20 have been shown to be implicated in the pathomechanism of preeclampsia, making CCL20 a potential inflammatory biomarker in this pathological condition [20]. Although the role of CCR6 has been investigated in relation to Th17/Treg cell subsets, its expression and potential relevance in γδT cells during pregnancy are still unclear. Taken together, these findings provide the rationale for examining the surface expression of CCR5 and CCR6 on circulating maternal γδT cell subsets during the first trimester of pregnancy, a period characterized by a tightly regulated pro-inflammatory immune environment and dynamic local and systemic immune adaptation. Such an analysis may expand the available evidence on chemokine receptor expression in this small but immunologically relevant T-cell population.

Current knowledge of ChR expression in γδT cells is largely based on studies examining expression patterns according to Vδ-chain usage, whereas the association between ChR expression and other phenotypic characteristics, including γδTCR intensity, CD56 expression, and CD4/CD8 co-expression, remains poorly defined. Although flow cytometric analysis can distinguish γδTCR^dim^ and γδTCR^bright^ γδT-cell subsets, it remains unclear whether these populations differ in their phenotypic characteristics and ChR profiles. Given that ChR expression contributes to cellular responsiveness to chemokine signals, peripheral blood analysis enables the assessment of subset-specific trafficking-associated receptor phenotypes in circulating immune cells. Therefore, the present study aimed to characterize the CD4/CD8 phenotypes and CCR5/CCR6 ChR profiles in circulating γδT-cell subsets defined by γδTCR intensity and in CD56-defined γδT-cell and non-γδT-cell populations, and to determine whether these profiles differ between healthy non-pregnant and first-trimester pregnant women. We further aimed to assess the effects of in vitro activation on these profiles.

## 2. Materials and Methods

### 2.1. Human Samples

Peripheral venous blood was obtained from healthy non-pregnant female donors (*n* = 29) at the Department of Medical Microbiology and Immunology, University of Pécs, Medical School, Hungary. Samples from healthy age-matched first-trimester pregnant women were collected at the Department of Obstetrics and Gynaecology, University of Pécs, Medical School, Hungary (*n* = 27; gestational age: 7–13 weeks). All female volunteers were healthy and selected to be free from chronic, autoimmune, or genital tract disorders. Individuals receiving hormone replacement therapy were also excluded. The study was conducted in accordance with the Declaration of Helsinki and was approved by the Regional Research Ethics Committee of the University of Pécs Clinical Centre (approval numbers 5643-PTE/2019 and 5643-PTE/2023). Written informed consent was obtained from all participants prior to their inclusion in the study.

### 2.2. Isolation of PBMCs

Ten milliliters of peripheral blood were collected from each participant and immediately diluted with 20 mL of phosphate-buffered saline (PBS) (Biosera, Nuaillé, France). The diluted blood samples were gently layered onto 10 mL of Ficoll-Paque™ solution (GE Healthcare Bio-Sciences AB, Uppsala, Sweden). Thereafter, peripheral blood mononuclear cells (PBMCs) were isolated by density gradient centrifugation. To remove any residual Ficoll, the PBMC layer was collected and washed twice with PBS. Subsequently, lymphocytes were stained with Türk’s solution and counted manually using a light microscope.

### 2.3. PBMC Preparation for Flow Cytometry

After PBMC isolation and counting, 1 × 10^6^ lymphocytes were incubated in PBS supplemented with 10% FBS for 4 h at 4 °C before antibody staining (baseline group). A similar number of cells was stimulated under antigen-independent conditions using PMA (phorbol myristate acetate) and Ionomycin to characterize ChR surface expression profiles under activation-associated experimental conditions. PMA acts as a diacylglycerol analogue that activates protein kinase C, whereas ionomycin increases intracellular Ca^2+^ levels. Together, they provide a strong, synchronized, and receptor-independent stimulus, allowing standardized comparison of CCR5/CCR6 regulation across the investigated lymphocyte subsets. Following counting, cells were transferred into fresh tubes and washed with PBS. The supernatant was discarded, and the cell pellet was resuspended in PBS supplemented with 10% FBS, ionomycin (1 µg/mL) (Sigma-Aldrich, St. Louis, MO, USA), and PMA (25 ng/mL) (Sigma-Aldrich) and incubated for 4 h at 37 °C in a humidified atmosphere containing 5% CO_2_. PMA and Ionomycin concentrations were selected based on previous protocols and earlier studies conducted by our group, as these concentrations were found to provide consistent and effective activation of γδT cells [21]. This activation was also shown to modulate ChR expression patterns [22].

### 2.4. Flow Cytometric Staining and Analysis

Following the 4 h incubation in both experimental settings, cells from each pretreatment condition were washed with PBS and stained for 25 min at room temperature in the dark using a 9-color flow cytometry panel consisting of nine fluorochrome-conjugated monoclonal antibodies (mAbs) (Sony Biotechnology Inc., San Jose, CA, USA), added simultaneously (Table 1). The cells were then washed with PBS, resuspended, and fixed in PBS containing 4% paraformaldehyde (PFA), and stored at 4 °C in the dark until flow cytometric analysis. Measurements were performed using a Navios™ flow cytometer (Beckman Coulter, Brea, CA, USA). Compensation matrices were calculated in FlowJo™ v9.8.5 (Tree Star, Inc., Ashland, OR, USA) using BD™ CompBeads (BD Biosciences, San Jose, CA, USA), and further analysis was performed using FCS Express v3 software (De Novo Software, Pasadena, CA, USA). To define marker-specific positivity thresholds, fluorescence minus one (FMO) controls were applied. The gating strategies used to identify the analyzed cell populations are presented in Supplementary Figures S1 and S2.

### 2.5. Statistics

Figures were created using GraphPad Prism 11, and statistical analyses were performed using IBM SPSS Statistics, version 27.0 (IBM Corp., Armonk, NY, USA). The Shapiro–Wilk test was applied to assess the normality of the data distribution prior to statistical analysis. For comparisons between two groups, an unpaired two-tailed Student’s *t*-test or the Mann–Whitney U test was used for normally and non-normally distributed data, respectively. The statistical significance among multiple subpopulations within one group was evaluated using one-way ANOVA, followed by Bonferroni’s post hoc correction. Comparisons between baseline and PMA/ionomycin-stimulated samples from the same individuals were performed using the paired *t*-test for normally distributed data and the Wilcoxon signed-rank test for non-normally distributed data. Differences were considered statistically significant at *p* ≤ 0.05.

## 3. Results

### 3.1. Distribution of γδTCR^dim^ and γδTCR^bright^ Subsets Among Resting and Recently Activated γδT Cells

**Figure 1:**
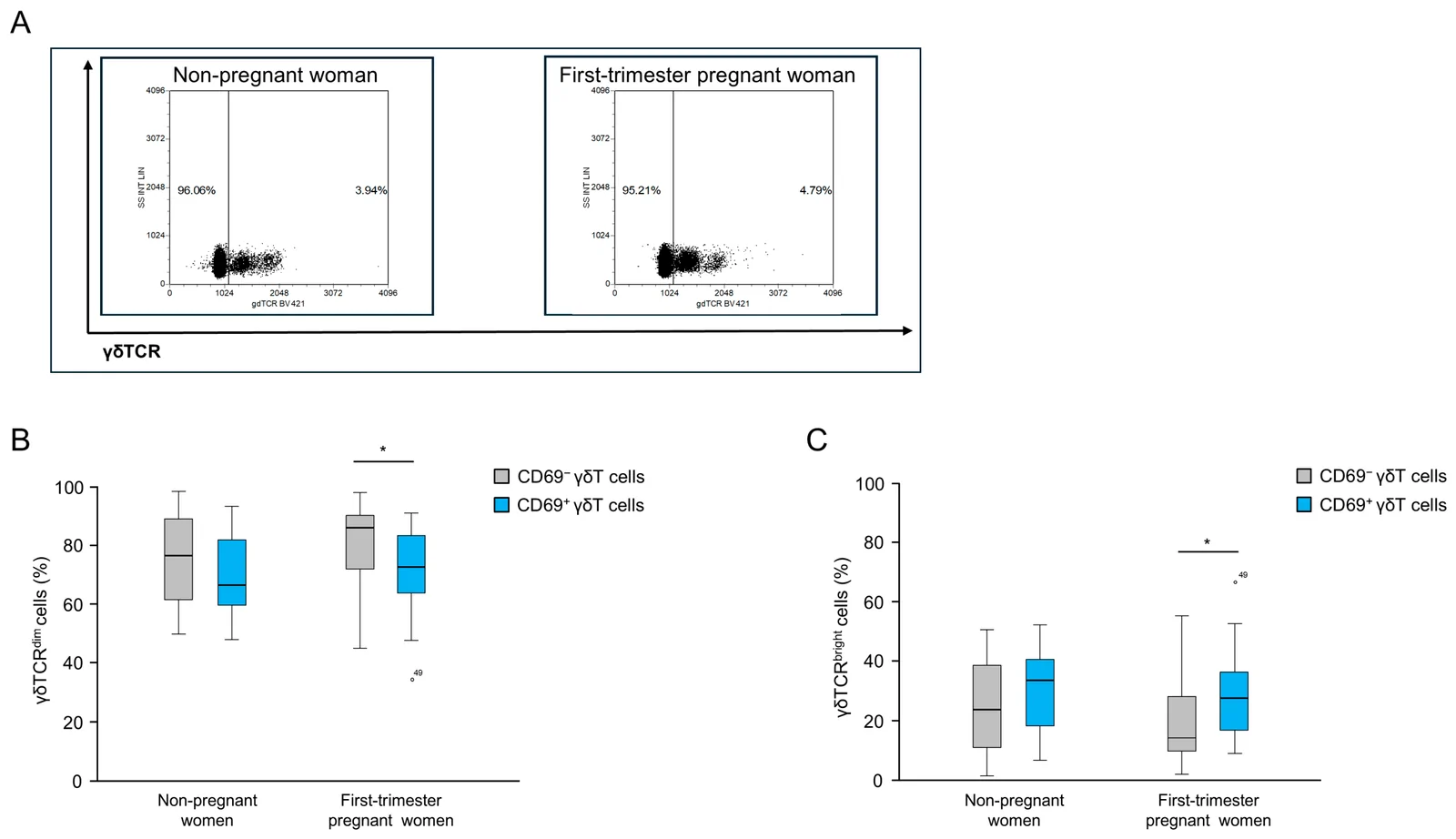
Distribution of γδTCR^dim^ and γδTCR^bright^ cell subsets in peripheral blood. (**A**) Representative FACS dot plots showing distinct γδTCR^dim^ and γδTCR^bright^ populations within the CD3^+^ gate from a non-pregnant and a first-trimester pregnant donor. (**B**) Frequencies of γδTCR^dim^ and (**C**) γδTCR^bright^ cells within resting (CD69^−^) and recently activated (CD69^+^) γδT cell gates in non-pregnant (*n* = 18) and first-trimester pregnant women (*n* = 23). Data are presented as box-and-whisker plots showing the median and interquartile range (IQR). Numbers next to isolated data points indicate SPSS case numbers for outliers. Normality was assessed using the Shapiro–Wilk test, and statistical comparisons between cell subsets or cohorts were performed using the unpaired two-tailed Student’s *t*-test (* *p* ≤ 0.05).

In the first part of our study, we investigated whether activation status, reflected by CD69 expression, influences the frequency of γδT cell subpopulations in freshly isolated PBMCs from non-pregnant and first-trimester pregnant individuals. CD69, an early activation marker [23], was used to distinguish resting (CD69^−^) and recently activated (CD69^+^) lymphocytes. Flow cytometric analysis revealed heterogeneity within γδT cells, identifying two subsets based on γδTCR expression intensity: γδTCR^dim^, which constituted the major population, and γδTCR^bright^, which represented a smaller fraction. Both subsets were consistently observed in most donor samples (Figure 1A). The distribution of these subsets was then assessed within resting and recently activated γδT cells. Our results showed that approximately 80% of CD69^−^ peripheral γδT cells were γδTCR^dim^, regardless of pregnancy, with a slightly but significantly lower proportion observed within CD69^+^ cells in the pregnant cohort (Figure 1B). By contrast, γδTCR^bright^ cells showed a significantly higher proportion within CD69^+^ than CD69^−^ cells during pregnancy (Figure 1C). When comparing non-pregnant and first-trimester pregnant cohorts, γδTCR^bright^ and γδTCR^dim^ cell proportions did not differ between cohorts in either CD69^−^ or CD69^+^ gates (Figure 1B,C).

### 3.2. Distinct Chemokine Receptor Profiles but Broadly Comparable Surface Marker Expression Distinguish γδTCR^dim^ and γδTCR^bright^ Cells

**Figure 2:**
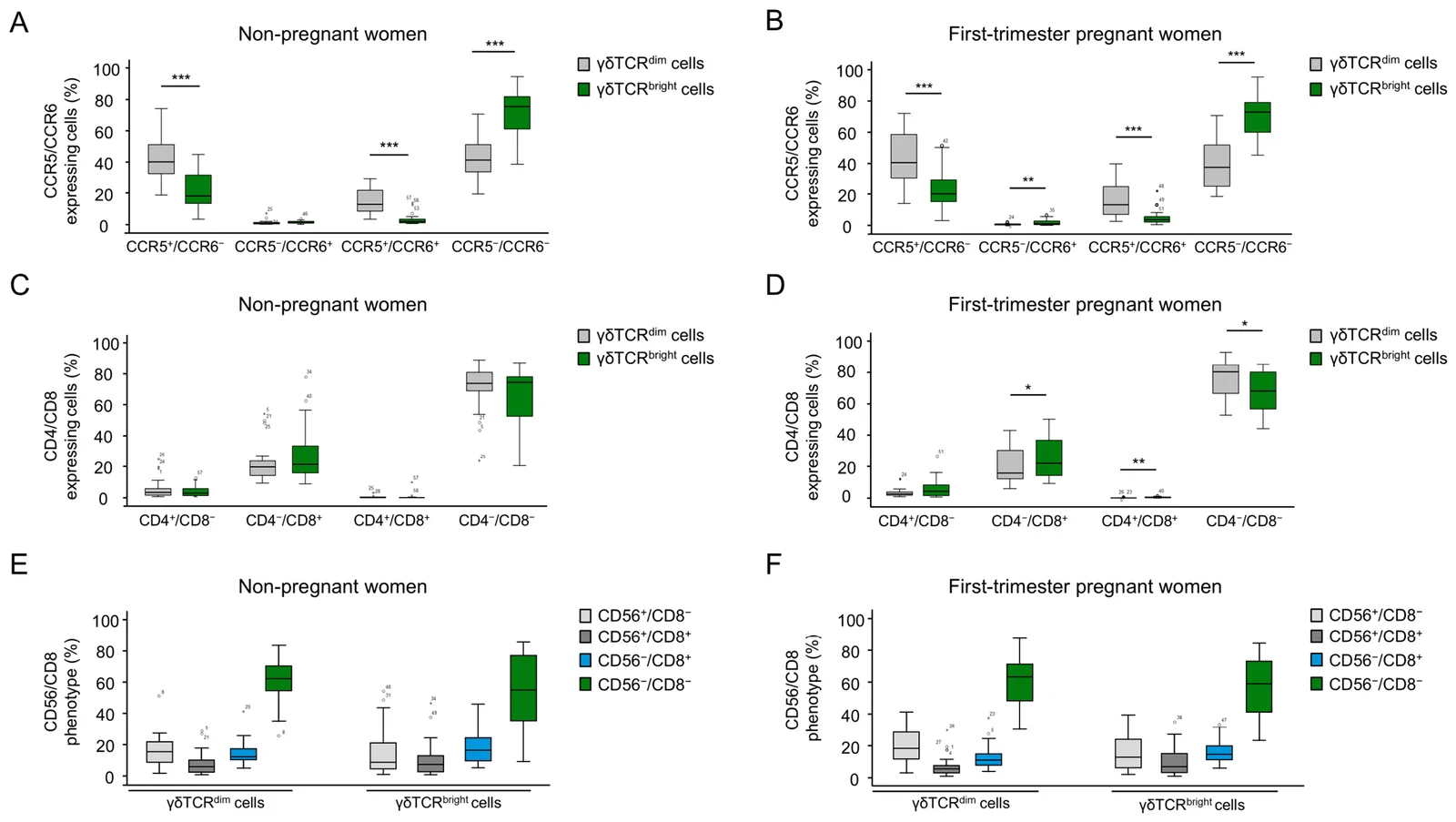
CCR5/CCR6 expression profiles, CD4/CD8, and CD56/CD8 co-expression patterns on resting γδTCR^dim^ and γδTCR^bright^ cell subsets in peripheral blood. (**A**,**B**) Frequencies of CCR5/CCR6-defined cell subsets within CD69^−^ γδTCR^dim^ and CD69^−^ γδTCR^bright^ cells in peripheral blood from (**A**) non-pregnant women and (**B**) first-trimester pregnant women. (**C**,**D**) Frequencies of CD4/CD8-defined cell subsets within CD69^−^ γδTCR^dim^ and CD69^−^ γδTCR^bright^ cells in peripheral blood from (**C**) non-pregnant women and (**D**) first-trimester pregnant women. (**E**,**F**) Dominance of CD56/CD8-defined cell subsets within CD69^−^ γδTCR^dim^ and CD69^−^ γδTCR^bright^ cells in peripheral blood from (**E**) non-pregnant women and (**F**) first-trimester pregnant women. Data are shown as box-and-whisker plots with the median and interquartile range (IQR). Numbers next to isolated data points indicate SPSS case numbers for outliers. Circles and asterisks are SPSS-generated symbols indicating outliers and extreme outliers. (*n* = 23 for both non-pregnant and pregnant donors across all panels, (**A**–**F**). Normality was assessed using the Shapiro–Wilk test. Comparisons between independent cell subsets or cohorts were performed using the unpaired two-tailed Student’s *t*-test or Mann–Whitney U test, as appropriate (* *p* ≤ 0.05, ** *p* ≤ 0.01, *** *p* ≤ 0.001). Statistical significance among the different phenotypes within each γδT cell subset was evaluated using one-way ANOVA followed by Bonferroni’s post hoc test.

Subsequently, we analyzed the phenotypic profiles of CD69^−^ γδTCR^dim^ and γδTCR^bright^ cells. Our analysis initially focused on the ChRs by identifying four distinct CCR5/CCR6 phenotypes: CCR5^+^/CCR6^−^, CCR5^−^/CCR6^+^, CCR5^+^/CCR6^+^, and CCR5^−^/CCR6^−^ subsets. We examined the predominance of these expression patterns and found that around 70% of γδTCR^bright^ cells lacked expression of both receptors (CCR5^−^/CCR6^−^), while this subset accounted for only about 40% of γδTCR^dim^ cells. CCR5 expression was consistently favored over CCR6 in both γδT cell subsets; however, this difference was more pronounced within the γδTCR^dim^ population. Notably, the prevalence of CCR5^+^/CCR6^−^ cells was markedly higher than that of CCR5^−^/CCR6^+^ or CCR5^+^/CCR6^+^ cells in both cohorts (*p* ≤ 0.001). By contrast, the frequency of CCR5^−^/CCR6^+^ cells was significantly lower than that of CCR5^+^/CCR6^+^ cells in γδTCR^dim^ cell subsets in both cohorts (*p* ≤ 0.001) (Figure 2A,B).

We next directly compared ChR expression profiles between γδTCR^bright^ and γδTCR^dim^ cells. The findings revealed that the latter exhibited approximately double the frequency of CCR5^+^/CCR6^−^ cells and a significantly higher proportion of CCR5^+^/CCR6^+^ cells compared with γδTCR^bright^ cells in both cohorts. In contrast, significantly higher frequencies of CCR5^−^/CCR6^+^ cells in pregnant women and of CCR5^−^/CCR6^−^ cells in both cohorts were observed among γδTCR^bright^ cells (Figure 2A,B).

Next, we characterized CD4 and CD8 expression patterns on both γδT cell subsets. The results showed highly similar distributions of CD4/CD8-defined subpopulations within the γδTCR^dim^ and γδTCR^bright^ compartments. As expected, CD4^−^/CD8^−^ cells were the most prevalent, followed by CD4^−^/CD8^+^, then CD4^+^/CD8^−^, and finally CD4^+^/CD8^+^ cells, which accounted for a negligible fraction. Direct comparisons between γδTCR^dim^ and γδTCR^bright^ cells revealed slight differences, which reached significance in the pregnant cohort only. Within this cohort, the proportions of CD4^−^/CD8^+^ cells and CD4^+^/CD8^+^ cells were significantly higher in the γδTCR^bright^ subpopulation, whereas γδTCR^dim^ cells exhibited a slightly but significantly elevated frequency of CD4^−^/CD8^−^ cells. However, a similar but non-significant trend was observed in the non-pregnant cohort (Figure 2C,D).

Finally, CD56/CD8 co-expression was assessed to determine whether γδTCR^dim^ and γδTCR^bright^ cells differed in the representation of cytotoxicity-associated NK-like phenotypes. The overall CD56/CD8 phenotypic patterns were comparable between γδT cell subsets and cohorts. In both γδT cell subsets, CD56^−^/CD8^−^ cells predominated, whereas CD56^+^/CD8^+^ cells represented the smallest population. The two single-positive populations, CD56^+^/CD8^−^ and CD56^−^/CD8^+^, were generally present at comparable frequencies (Figure 2E,F).

Comparisons between the non-pregnant and pregnant cohorts revealed no significant differences in CCR5/CCR6, CD4/CD8, or CD56/CD8 co-expression patterns in either γδT cell population (Figure 2).

### 3.3. Early Activation-Associated Distribution of CD4/CD8 and CD56-Defined NK-like Phenotypes in Non-γδT and γδT Cells

We then extended our analysis to the total γδT and non-γδT populations within resting and recently activated peripheral blood lymphocytes. γδT cells were identified based on γδTCR expression within the CD3^+^ lymphocyte gate, whereas γδTCR^−^ cells served as the non-γδT control group (primarily representing αβT cells). After defining these populations, we evaluated their distribution, as well as CD4/CD8 and CD56 expression profiles within resting and recently activated compartments in non-pregnant and pregnant cohorts.

**Figure 3:**
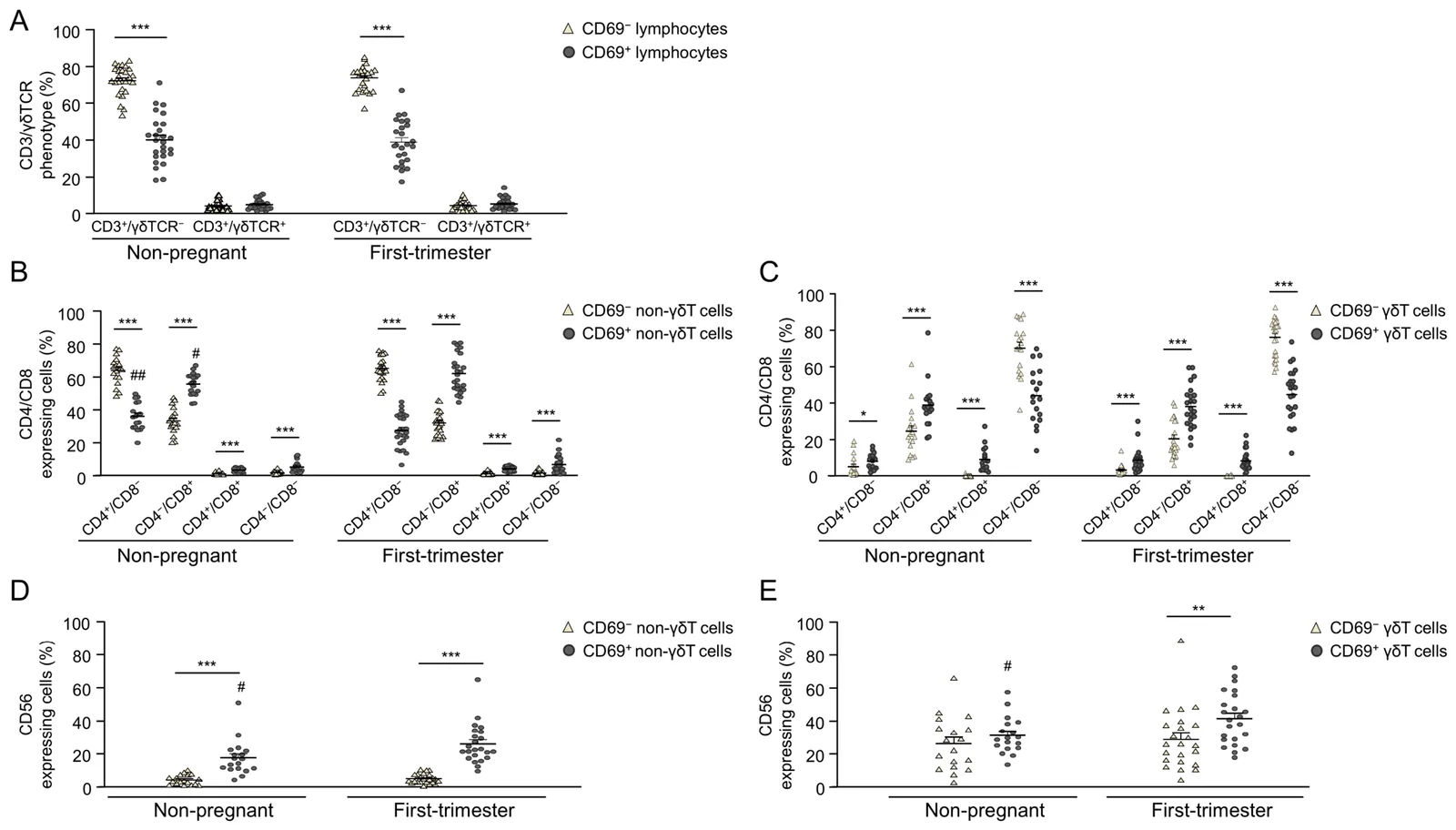
Distribution of peripheral blood non-γδT and γδT cells and their CD4/CD8- and CD56-defined phenotypes among resting and recently activated subsets in non-pregnant and first-trimester pregnant women. (**A**) Frequency of CD3/γδTCR-defined lymphocyte cell subsets within resting (CD69^−^) and recently activated (CD69^+^) lymphocytes in peripheral blood from non-pregnant women (*n* = 26) and first-trimester pregnant women (*n* = 25). (**B**,**C**) Frequency of CD4/CD8-defined subsets within resting (CD69^−^) and recently activated (CD69^+^) T cells in peripheral blood from non-pregnant women (*n* = 18) and first-trimester pregnant women (*n* = 23): (**B**) non-γδT cells and (**C**) γδT cells. (**D**,**E**) Frequency of CD56^+^ cell subsets within resting (CD69^−^) and recently activated (CD69^+^) T cells in peripheral blood from non-pregnant women (*n* = 18) and first-trimester pregnant women (*n* = 23): (**D**) non-γδT cells and (**E**) γδT cells. Data are shown as mean with upper SEM. Individual data points are represented as triangles (CD69^−^) or circles (CD69^+^). The Shapiro–Wilk test was used to evaluate data normality, and comparisons between independent cell subsets or cohorts were conducted using the unpaired two-tailed Student’s *t*-test or Mann–Whitney U test, as appropriate (* *p* ≤ 0.05, ** *p* ≤ 0.01, *** *p* ≤ 0.001). Hash symbols placed above the respective non-pregnant data sets denote statistically significant differences between the non-pregnant and first-trimester pregnant cohorts for the same CD4/CD8 or CD56 phenotype within the indicated T-cell subset (^#^ *p* ≤ 0.05, ^##^ *p* ≤ 0.01). Statistical significance across multiple phenotypes within each T cell subset was evaluated using one-way ANOVA followed by Bonferroni’s post hoc correction.

Our results revealed distinct activation-associated distributions within the CD45^+^ lymphocyte population. Non-γδT cells predominantly exhibited a resting phenotype, with markedly higher frequencies in the CD69^−^ fraction than in the CD69^+^ fraction across both cohorts (Figure 3A). By contrast, despite their low baseline frequency in peripheral blood, γδT cells showed similar proportions between the CD69^−^ and CD69^+^ gates in both cohorts (Figure 3A).

Analysis of CD4/CD8 co-receptor expression alongside NK-associated phenotypic features revealed significant differences between the two T cell types. Among non-γδT cells, the dominant CD4^+^/CD8^−^ and CD4^−^/CD8^+^ populations exhibited activation-dependent distributions; CD4^+^/CD8^−^ cells were enriched in the resting fraction, whereas CD4^−^/CD8^+^ cells were more prevalent in the recently activated fraction across both cohorts. In contrast, γδT cells were predominantly CD4^−^/CD8^−^, particularly within the CD69^−^ population (*p* ≤ 0.001), as they were notably more abundant within the resting pool (Figure 3B,C).

Direct comparison of resting and recently activated states revealed that early activation altered CD4/CD8 co-receptor profiles in a T cell subset-specific manner. Activation was associated with increased frequencies of CD4^−^/CD8^+^ and CD4^+^/CD8^+^ cells in both T cell subsets. In contrast, CD4^+^/CD8^−^ and CD4^−^/CD8^−^ populations exhibited reciprocal trends between non-γδT and γδT cells (Figure 3B,C).

To assess NK-like phenotypic features, we analyzed CD56 expression, which was substantially higher in γδT cells than in non-γδT cells, accounting for approximately 27% versus ~5% of the respective populations within the CD69^−^ gate (*p* ≤ 0.001). Comparison of resting and recently activated fractions revealed that the frequency of CD56^+^ γδT cells was significantly increased within the CD69^+^ fraction in the pregnant cohort only, whereas CD56^+^ non-γδT cells were consistently more frequent in the CD69^+^ fraction across both cohorts (Figure 3D,E).

Finally, we compared the pregnant and non-pregnant cohorts and found that significant pregnancy-associated differences were restricted to the recently activated (CD69^+^) compartment. While the distribution of CD4/CD8-defined γδT cell subsets remained similar between cohorts, activated non-γδT cells exhibited pregnancy-associated shifts characterized by reduced CD4^+^/CD8^−^ and increased CD4^−^/CD8^+^ cell frequencies. Furthermore, the frequency of NK-like T cells was elevated during pregnancy; within the recently activated population, both CD56^+^ γδT and CD56^+^ non-γδT cells were more frequent in pregnant women (Figure 3).

### 3.4. Subset-Specific CCR5/CCR6 Profiles of Conventional Non-γδT and γδT Cells Under Baseline and PMA/Ionomycin-Stimulated Conditions

We next evaluated CCR5/CCR6 expression profiles across CD56^−^ conventional non-γδT and γδT cell subsets classified by CD4/CD8 phenotype. CD4^+^/CD8^+^ double-positive populations were excluded due to their extremely low frequency. To investigate pregnancy-associated differences in ChR expression, analyses were performed in both women cohorts under baseline and PMA/ionomycin-stimulated conditions.

**Figure 4:**
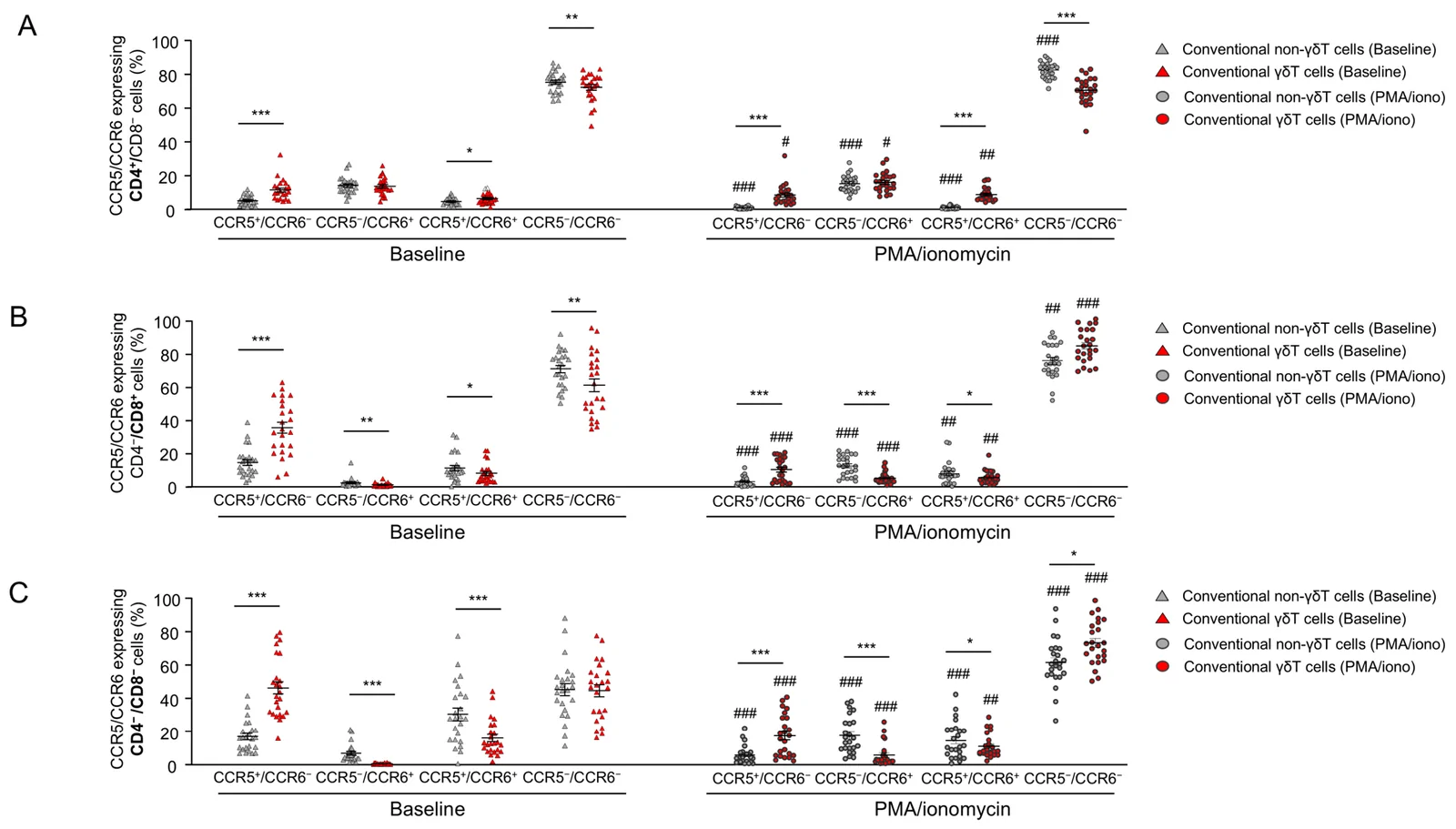
CCR5/CCR6 expression profiles of peripheral blood conventional non-γδT (CD56^−^/γδTCR^−^) and conventional γδT (CD56^−^/γδTCR^+^) cell subsets defined by CD4/CD8 expression in first-trimester pregnant women. Frequencies of CCR5/CCR6-defined cell subsets were assessed within CD4/CD8 populations of conventional non-γδT (CD56^−^/γδTCR^−^) and conventional γδT cells (CD56^−^/γδTCR^+^) within CD3^+^ lymphocytes in peripheral blood from pregnant women under baseline and PMA/ionomycin-stimulated conditions. CD4^+^/CD8^+^ populations were excluded due to insufficient event counts. (**A**) Frequencies of CCR5/CCR6-defined cell subsets within the CD4^+^/CD8^−^ population under baseline (CD69^−^/CD4^+^/CD8^−^) and PMA/ionomycin-stimulated conditions (CD69^+^/CD4^+^/CD8^−^). (**B**) Frequencies of CCR5/CCR6-defined cell subsets within the CD4^−^/CD8^+^ population under baseline (CD69^−^/CD4^−^/CD8^+^) and PMA/ionomycin-stimulated conditions (CD69^+^/CD4^−^/CD8^+^). (**C**) Frequencies of CCR5/CCR6-defined cell subsets within the CD4^−^/CD8^−^ population under baseline (CD69^−^/CD4^−^/CD8^−^) and PMA/ionomycin-stimulated conditions (CD69^+^/CD4^−^/CD8^−^). Data from first-trimester pregnant women (*n* = 24) are shown as mean ± SEM. The individual data points are represented as triangles (baseline) or circles (PMA/ionomycin stimulated). Normality was assessed using the Shapiro–Wilk test. Statistical significance across multiple phenotypes within each T cell subset was evaluated using one-way ANOVA followed by Bonferroni’s post hoc correction. Comparisons between independent subsets and cohorts were performed using the unpaired two-tailed Student’s *t*-test or Mann–Whitney U test, as appropriate. Comparisons between paired groups were performed using the paired Student’s *t*-test for normally distributed data or the Wilcoxon signed-rank test for non-normally distributed data. Statistical significance is indicated as * *p* ≤ 0.05, ** *p* ≤ 0.01, *** *p* ≤ 0.001. Hash symbols placed above the PMA/ionomycin-activated data indicate significant differences from baseline within the same T-cell subset and CCR5/CCR6 phenotype (^#^ *p* ≤ 0.05, ^##^ *p* ≤ 0.01, ^###^ *p* ≤ 0.001).

Distinct CCR5/CCR6 expression profiles were observed across the investigated conventional T cell compartments. Within the CD4^+^/CD8^−^ population, CCR5^−^/CCR6^−^ cells predominated in both conventional T cell subsets regardless of pregnancy or activation status (*p* ≤ 0.001), while all remaining CCR5/CCR6 phenotypes, including the second most abundant CCR5^−^/CCR6^+^ phenotype, were present only at substantially lower frequencies (Figure 4A and Figure S3A). During pregnancy, CCR5^−^/CCR6^−^ cells were slightly but significantly more frequent among conventional non-γδT cells than γδT cells. In contrast, CCR5-expressing cells, despite representing only minor populations, were consistently more abundant in conventional γδT cells (Figure 4A and Figure S3A).

The CD4^−^/CD8^+^ and CD4^−^/CD8^−^ subpopulations of conventional non-γδT and γδT cells displayed broadly similar ChR distribution patterns regardless of cohort, although CCR5^−^/CCR6^−^ predominance was less pronounced in the CD4^−^/CD8^−^ phenotype. Unlike in the CD4^+^/CD8^−^ subset, CCR5^+^/CCR6^−^ cells represented the second most abundant population in both CD4^−^ conventional γδT-cell subsets, namely CD4^−^/CD8^+^ and CD4^−^/CD8^−^ conventional γδT cells. This pattern was particularly pronounced in CD4^−^/CD8^−^ γδT cells, where CCR5^−^/CCR6^−^ and CCR5+/CCR6^−^ cells were equally represented. Compared with conventional non-γδT cells, conventional γδT cells exhibited higher CCR5^+^/CCR6^−^ frequencies and lower frequencies of CCR6-expressing phenotypes across both CD4^−^/CD8^+^ and CD4^−^/CD8^−^ compartments, particularly during pregnancy, whereas lower CCR5^−^/CCR6^−^ frequencies were observed in the CD4^−^/CD8^+^ subset (Figure 4B,C and Figure S3B,C).

Furthermore, CCR5/CCR6 expression profiles were also evaluated in PMA/ionomycin-stimulated samples. Within the activated cohorts, CCR5^+^/CCR6^−^ cells represented low proportions in all CD4/CD8-defined conventional T cell subsets, while remaining more frequent in conventional γδT cells than in non-γδT cells. The most pronounced differences between baseline and stimulated groups were observed within the CD4^−^/CD8^+^ and CD4^−^/CD8^−^ conventional T cell subsets of both cohorts, where stimulated samples exhibited significantly lower CCR5^+^/CCR6^−^ frequencies. Similarly, CCR5^+^/CCR6^+^ cells were less frequent under stimulated conditions, particularly in the CD4^−^/CD8^−^ compartment. In contrast, the frequencies of CCR5^−^/CCR6^+^ cells were just slightly higher in the CD4^+^/CD8^−^ compartment of stimulated samples, whereas much higher in both CD4^−^/CD8^+^ and CD4^−^/CD8^−^ populations among both conventional T cell subsets. In stimulated samples, CCR5^−^/CCR6^−^ cells remained the dominant population and were generally present at higher frequencies than in baseline samples, particularly within CD4^−^/CD8^−^ conventional T cells (Figure 4 and Figure S3).

Regarding pregnancy status, CCR5/CCR6 expression profiles were highly comparable between samples from non-pregnant and pregnant women across all investigated cell populations. The only exception was observed under baseline conditions, where CD4^+^/CD8^−^ conventional non-γδT cells from pregnant women exhibited a slightly but significantly lower frequency of CCR5^+^/CCR6^+^ cells (*p* ≤ 0.05).

### 3.5. CCR5/CCR6 Co-Expression Profiles Are Preserved in NK-like γδT Cells but Differ in NK-like Non-γδT Cells

We next extended the CCR5/CCR6 co-expression analysis to CD56^+^ non-γδT and γδT cell subsets. Given the association of CD56 expression with innate-like phenotype and distinct functional properties, including migratory potential, these populations were analysed separately. CD56^+^/γδTCR^−^ cells were defined as NK-like non-γδT cells, whereas CD56^+^/γδTCR^+^ cells were designated as NK-like γδT cells. Both subsets were further classified according to CD4 and CD8 expression; however, CD4^+^/CD8^−^ and CD4^+^/CD8^+^ populations were excluded from subsequent analyses because of the insufficient event counts.

**Figure 5:**
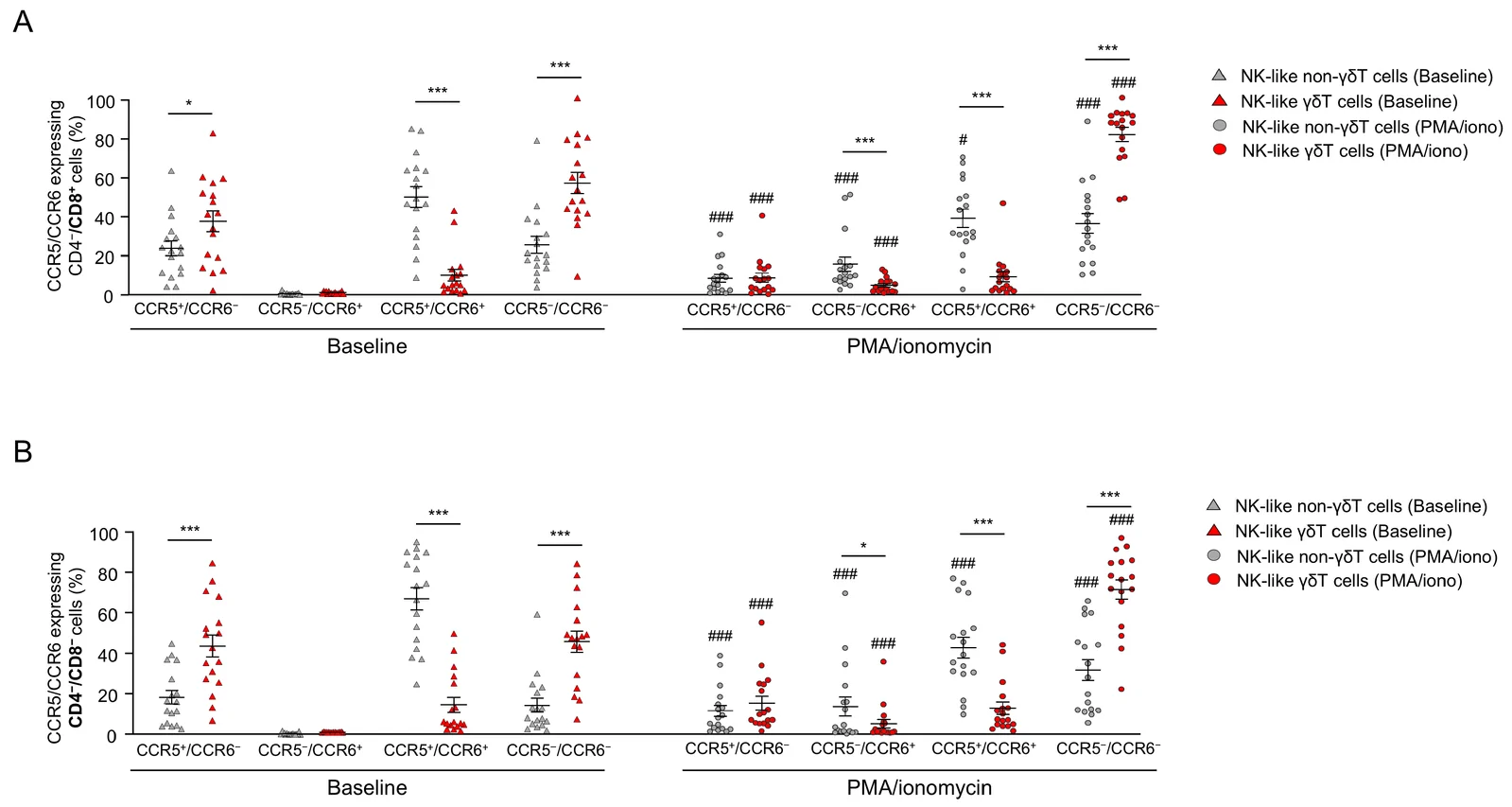
CCR5/CCR6 expression profiles of peripheral blood NK-like non-γδT (CD56^+^/γδTCR^−^) and NK-like γδT (CD56^+^/γδTCR^+^) cell subsets defined by CD4/CD8 expression in first-trimester pregnant women. Frequencies of CCR5/CCR6-defined cell subsets were assessed within CD4/CD8 populations of NK-like non-γδT (CD56^+^/γδTCR^−^) and NK-like γδT (CD56^+^/γδTCR^+^) cells within CD3^+^ lymphocytes in peripheral blood from pregnant women under baseline and PMA/ionomycin-stimulated conditions. CD4^+^/CD8^−^ and CD4^+^/CD8^+^ populations were excluded owing to insufficient event counts. (**A**) Frequencies of CCR5/CCR6-defined cell subsets within the CD4^−^/CD8^+^ population under baseline (CD69^−^/CD4^−^/CD8^+^) and PMA/Ionomycin stimulated conditions (CD69^+^/CD4^−^/CD8^+^). (**B**) Frequencies of CCR5/CCR6-defined cell subsets within the CD4^−^/CD8^−^ population under baseline (CD69^−^/CD4^−^/CD8^−^) and PMA/Ionomycin stimulated conditions (CD69^+^/CD4^−^/CD8^−^). Data from first-trimester pregnant women (*n* = 17), are shown as mean ± SEM. The individual data points are represented as triangles (baseline) or circles (PMA/ionomycin stimulated). Normality was assessed using the Shapiro–Wilk test. Statistical significance across multiple phenotypes within each T cell subset was evaluated using one-way ANOVA followed by Bonferroni’s post hoc correction. Comparisons between independent subsets and cohorts were performed using the unpaired two-tailed Student’s *t*-test or Mann–Whitney U test, as appropriate. Comparisons between paired groups were performed using the paired Student’s *t*-test or the Wilcoxon signed-rank test, according to data normality. Statistical significance is indicated as * *p* ≤ 0.05, *** *p* ≤ 0.001. Hash symbols placed above the PMA/ionomycin-activated data indicate significant differences from baseline within the same T-cell subset and CCR5/CCR6 phenotype (^#^ *p* ≤ 0.05, ^###^ *p* ≤ 0.001).

Under baseline conditions, patterns were broadly comparable between pregnant and non-pregnant cohorts. NK-like γδT and NK-like non-γδT cells displayed several CCR5/CCR6 co-expression characteristics similar to those observed in their conventional counterparts, including higher CCR5^+^/CCR6^−^ and lower CCR5^+^/CCR6^+^ frequencies in γδT than in non-γδT cells across both analyzed CD4/CD8-defined subsets. By contrast, CCR5^−^/CCR6^+^ phenotypes were negligible in all investigated CD4/CD8-defined NK-like T cell populations. In the CD4^−^/CD8^+^ and CD4^−^/CD8^−^ subsets, CCR5^−^/CCR6^−^ frequencies were significantly higher in NK-like γδT than in NK-like non-γδT cells in both cohorts, whereas the corresponding conventional subsets showed the opposite relationship detected in the CD4^−^/CD8^+^ compartment only (Figure 5 and Figure S4).

Following PMA/ionomycin stimulation, the relative distributions of the CCR5^+^/CCR6^+^ and CCR5^−^/CCR6^−^ phenotypes between NK-like γδT and NK-like non-γδT cells were largely consistent with those observed in conventional T cell subsets, except in the CD4^−^/CD8^+^ subset, where CCR5^−^/CCR6^−^ frequencies were higher in NK-like γδT than in NK-like non-γδT cells. However, CCR5^−^/CCR6^+^ cells were less frequent in NK-like γδT than in NK-like non-γδT cells across both analyzed CD4/CD8-defined subsets, whereas CCR5^+^/CCR6^−^ frequencies remained comparable between the two NK-like T cell populations, particularly during pregnancy (Figure 5 and Figure S4).

Compared with baseline samples, PMA/ionomycin-stimulated samples showed significantly lower CCR5^+^/CCR6^−^ frequencies and significantly higher CCR5^−^/CCR6^−^ frequencies in both NK-like T cell populations across both cohorts. In addition, CCR5^−^/CCR6^+^ frequencies were higher in stimulated samples of the pregnant cohort. Although CCR5^+^/CCR6^+^ frequencies were comparable among NK-like γδT cells, lower frequencies were observed among NK-like non-γδT cells following stimulation in both cohorts (Figure 5 and Figure S4).

Direct comparison of conventional and NK-like γδT cells revealed highly similar CCR5/CCR6 co-expression profiles between the two γδT cell populations. The only statistically significant difference observed was confined to the CD4^−^/CD8^−^ subset, where CCR5^−^/CCR6^+^ cells were more frequent in conventional γδT cells, particularly under baseline conditions, in both cohorts, although their frequencies remained very low, below 1% in both populations (*p* ≤ 0.001) (Figure 4, Figure 5 and Figures S3 and S4).

By contrast, more pronounced differences emerged between conventional and NK-like non-γδT cells. The CCR5^+^/CCR6^+^ phenotype was markedly more frequent in NK-like non-γδT cells, whereas CCR5^−^/CCR6^−^ cells were more prevalent in conventional non-γδT cells across both analyzed CD4/CD8-defined subsets, cohorts, and conditions (*p* ≤ 0.001). The CCR5/CCR6 single-positive phenotypes showed subset- and condition-dependent patterns. At baseline, CCR5^+^/CCR6^−^ cells were detected at higher frequencies in NK-like non-γδT cells only within the CD4^−^/CD8^+^ subset in both cohorts (*p* ≤ 0.05). In PMA/ionomycin-stimulated samples, this difference was present in the two CD4/CD8-defined subsets across both cohorts (*p* ≤ 0.01 for the CD4^−^/CD8^+^ subsets, and *p* ≤ 0.05 for the CD4^−^/CD8^−^ subsets). Conversely, CCR5^−^/CCR6^+^ cells were more frequent in conventional non-γδT cells at baseline in both CD4/CD8-defined subsets of both cohorts (*p* ≤ 0.001), although their frequencies remained low. In the stimulated samples, this pattern persisted only in the CD4^−^/CD8^−^ subset (*p* ≤ 0.05 in the pregnant cohort, and *p* ≤ 0.001 in the non-pregnant cohort) (Figure 4, Figure 5 and Figures S3 and S4).

Finally, no significant differences in CCR5/CCR6 co-expression profiles were detected between pregnant and non-pregnant women in any investigated NK-like subset under baseline or stimulated conditions (Figure 5 and Figure S4).

## 4. Discussion

This study characterized the phenotypic organization and CCR5/CCR6 co-expression profiles of circulating γδT cells in healthy non-pregnant and first-trimester pregnant women. Analysis of γδTCR expression intensity, CD69 status, CD4/CD8 and CD56 expression, and CCR5/CCR6 phenotypes identified three principal findings. First, γδTCR^dim^ and γδTCR^bright^ cells represented reproducible circulating γδT-cell subsets that differed primarily in their CCR5/CCR6 co-expression profiles, whereas their CD4/CD8 and CD56/CD8 distributions were largely comparable. Second, γδT and non-γδT cells exhibited distinct phenotypic organization within resting and recently activated compartments. Third, CCR5/CCR6 co-expression profiles were broadly maintained between conventional and NK-like γδT cells but showed greater phenotypic divergence between conventional and NK-like non-γδT cells. Together, these findings indicate that γδT-cell heterogeneity in peripheral blood is reflected more strongly in ChR organization than in broad co-receptor or NK-like surface-marker patterns. Overall, healthy first-trimester pregnancy was not associated with consistent or major alterations in the peripheral immune phenotypes examined.

We focused on peripheral blood because it provides an accessible compartment for investigating whether pregnancy-associated immune adaptation is accompanied by systemic changes in circulating lymphocyte phenotypes. It also offers insight into the migration-associated receptor profiles of maternal immune cells, including whether these profiles are influenced by pregnancy. However, peripheral blood is not equivalent to the decidual microenvironment, where a complex immune network of trophoblast-derived signals, stromal cells, cytokines, and chemokines shapes immune-cell differentiation and function. Therefore, the present findings should primarily be interpreted as features of circulating immune cells during first-trimester pregnancy rather than as direct evidence of decidual immune mechanisms. This distinction is important, because stable peripheral phenotypes do not exclude pregnancy-related immune adaptation at the maternal–fetal interface.

The consistent identification of γδTCR^dim^ and γδTCR^bright^ cells across donors supports the heterogeneous nature of human γδT cells. Although the overall distribution of the two subsets remained remarkably similar across the pregnant and non-pregnant study groups, a modest but significant shift in their relative representation between resting and recently activated γδT cells was observed during pregnancy. Because this difference was modest, it likely reflects subtle variation in the relative distribution of the two subsets rather than a major shift in the γδTCR^dim^/γδTCR^bright^ balance. Whether this difference reflects distinct activation dynamics or intrinsic biological differences between the subsets remains to be determined.

Beyond their activation-associated distribution, the most pronounced distinction between γδTCR^dim^ and γδTCR^bright^ cells was observed in their CCR5/CCR6 co-expression profiles. γδTCR^dim^ cells showed higher frequencies of CCR5 single-positive and CCR5/CCR6 double-positive cells, whereas the majority of γδTCR^bright^ cells displayed a double-negative CCR5/CCR6 phenotype. Since CCR5 is a Th1 marker, commonly expressed on activated and effector memory T cells with inflammatory-site homing potential [24], the preferential CCR5 expression observed in the γδTCR^dim^ subset may reflect greater migratory potential toward inflammatory tissues producing CCL3, CCL4, and CCL5, the main CCR5 ligands. Overall, the biological relevance of these γδTCR intensity-defined phenotypic differences remains to be clarified. Our group previously reported higher TIM-1 expression on γδTCR^bright^ than on γδTCR^dim^ cells in murine decidual models, suggesting that γδTCR^bright^ cells may contribute to the pregnancy-associated Th2/tolerogenic environment [25]. Although no functional assays were performed in the present study, the observed phenotypic differences raise the possibility that γδTCR^dim^ and γδTCR^bright^ cells differ not only in TCR expression intensity but also in biological properties. In this regard, γδT cell subsets may show conceptual similarity to the well-established CD56^dim^ and CD56^bright^ NK-cell subsets, in which phenotypic differences are associated with distinct functional characteristics [26].

By contrast, the two γδT cell subsets displayed broadly similar CD4/CD8 distributions, with cells lacking both co-receptors representing the predominant population. The general predominance of this phenotype is consistent with our current knowledge about peripheral γδT cells [8]. Additionally, CD56/CD8 co-expression profiles were also largely similar between the subsets. This observation is in line with our previous data in a murine model, where the expression of CD160, an NK cell-associated receptor that is linked to cytotoxic function, was comparable between γδTCR^dim^ and γδTCR^bright^ cells in the decidua [25]. Collectively, these findings suggest that γδTCR expression intensity is more closely associated with ChR expression patterns than with co-receptor expression or NK-like phenotypic characteristics. Because CCR5 and CCR6 are involved in lymphocyte trafficking, these differences may reflect distinct migration-associated states. Immune cell migration is a highly dynamic process regulated by multiple factors, including adhesion molecules and endothelial activation status [27]. Therefore, assessment of ChR expression alone cannot establish chemotactic capacity or tissue homing. Functional migration assays will also be required to determine whether these phenotypic differences translate into distinct migratory responses.

Having considered heterogeneity within the γδT cell compartment, we next compared γδT cells with the non-γδT cell population. Non-γδT cells were defined as γδTCR^−^ cells, primarily representing the conventional αβT-cell population, accounting for approximately 95% of CD3^+^ peripheral blood lymphocytes, whereas γδT cells represented approximately 5%. Comparison of resting and recently activated lymphocytes further showed that γδT and non-γδT cells displayed distinct distribution patterns across the CD69-defined lymphocyte compartments. Because CD69 is an early activation marker that is transiently expressed and subsequently downregulated after activation [28], these observations suggest that recently activated γδT and non-γδT cells show cell-population-specific patterns of phenotypic organization, consistent with their different functional roles. In addition, investigating complementary activation markers, such as CD25, might provide deeper insight into more sustained activation states.

The two T cell populations also exhibited different CD4/CD8 phenotypes, a finding consistent with their known biological identities. Regarding NK-like characteristics, available data on CD56 expression by γδT cells during pregnancy remain limited. A prior study by our group described an increased prevalence of NK-like γδT cells in peripheral blood during the second half of human pregnancy [8]. The results of the present study showed that CD56 expression was more frequent among γδT than non-γδT cells, supporting the concept that circulating γδT cells include a larger NK-like component than classical αβT cell populations. Given that CD56 is typically associated with innate-like and potentially cytotoxic phenotypes, its higher prevalence among peripheral γδT cells is consistent with their known NK-like functional features [7]. It is important to note that, although CD56 expression indicates an NK-like phenotype, it should not be interpreted as evidence of cytotoxic activity. Thus, CD56 expression should be regarded here as a phenotypic marker of NK-like differentiation rather than as a functional readout. Overall, these observations further emphasize the phenotypic distinction between circulating γδT and non-γδT cells and provide a framework for the subsequent comparison of their ChR expression profiles.

During pregnancy, the majority of peripheral and decidual γδT cells display a CD4^−^/CD8^−^ phenotype, although CD4^+^ and CD8^+^ subpopulations are also present at lower frequencies [8,29]. However, ChR expression profiles of peripheral conventional CD56^−^ γδT cells classified by CD4/CD8 co-expression, as well as their relationship to NK-like CD56^+^ γδT cell subsets, had not been characterized in this setting. Therefore, the next step of our analysis was to determine whether the differences observed between γδT and non-γδT cells were also reflected in their ChR expression patterns. Our analysis first focused on conventional CD56^−^ T cell populations to exclude the potential influence of NK-like phenotypes. CCR5/CCR6 co-expression patterns differed according to both T cell subset and CD4/CD8 phenotype, with CCR5^−^/CCR6^−^ cells predominating in most CD4/CD8-defined conventional T cell compartments. However, conventional γδT cells differed from conventional non-γδT cells by showing a greater representation of CCR5-expressing phenotypes and a lower representation of CCR6-expressing phenotypes, particularly within CD4^−^ subsets. These differences may reflect the distinct biological characteristics of γδT and non-γδT cells, which may also shape their ChR expression patterns. This pattern indicates that γδT and non-γδT cells are not only separated by their co-receptor composition but also by their ChR organization. Our findings align with previous studies showing differences in CCR5 expression between γδT and αβT cells [30].

In contrast to CD4^−^ conventional T cell subsets, where the CCR5^+^/CCR6^−^ phenotype represented the second most abundant population, particularly among γδT cells, this phenotype was only modestly represented in the CD4^+^ γδT cell subset. These results suggest that CD4 expression could be an important determinant of CCR5/CCR6 organization within conventional T cells, rather than merely a marker used for subgrouping. This interpretation is important because it places CD4/CD8 expression into a broader phenotypic context: in this dataset, co-receptor status appears to be linked to ChR patterning. The overall CCR5/CCR6 distribution patterns across CD4/CD8-defined T cell subsets were largely retained under the two experimental conditions, supporting their relative stability within both conventional γδT and conventional non-γδT cell compartments. Nonetheless, it remains to be determined whether the higher proportion of CCR6-expressing cells within the CD4^+^/CD8^−^ γδT cell subset, together with the preferential CCR5 expression observed on the CD4^−^/CD8^−^ γδT cell subset, reflects differences in trafficking capacity, differentiation status, or both. Future studies integrating ChR profiling with memory differentiation markers, such as CD45RO, may help clarify the biological basis of these phenotypic differences found among CD4/CD8-defined subsets.

Current evidence suggests that in activated immune cells, ChR expression profiles can be modulated, thereby altering tissue homing potential [31,32]. In our study, PMA/ionomycin-stimulated samples were characterized by a lower representation of CCR5^+^ phenotypes and a higher representation of CCR5^−^ phenotypes in several conventional T cell subsets compared with baseline samples. The observed condition-related differences in ChR expression may be related, at least in part, to PMA-induced activation of protein kinase C (PKC), a signaling pathway known to influence ChR expression patterns [33]. This established link supports the use of PMA to assess the sensitivity of ChR expression to activation, while ionomycin complements PMA by engaging Ca^2+^-dependent signaling. Together, they activate two major downstream pathways involved in lymphocyte activation. Although PMA/ionomycin bypasses physiological receptor engagement and does not reproduce the complex hormonal, cytokine, and receptor-specific signals acting on circulating immune cells in vivo during pregnancy, this strong and synchronized stimulus enables standardized comparison of activation-associated ChR regulation across lymphocyte subsets. Accordingly, our findings should not be interpreted as providing a direct model of the physiological activation of circulating immune cells in vivo. Instead, this approach provided a standardized framework for assessing how CCR5/CCR6 organization responds to activation across lymphocyte subsets and whether this responsiveness differs between samples from pregnant and non-pregnant women. Within this experimental context, the low CCR5^+^ cell proportions detected in stimulated samples may suggest lower CCR5-associated inflammatory homing potential. Notably, pregnancy-associated differences remained minimal and were limited to a slight change within the CD4^+^ conventional non-γδT cell compartment, suggesting no broader biological relevance. In the following section, these findings in conventional T cells provide an important reference point for interpreting the characteristics of the NK-like T cell compartment.

We next examined whether CD56 expression was associated with different CCR5/CCR6 profiles in γδT and non-γδT cells. The most notable finding was that conventional and NK-like γδT cells displayed highly similar CCR5/CCR6 co-expression profiles, whereas conventional and NK-like non-γδT cells showed pronounced differences. These findings suggest that acquisition of an NK-like phenotype is associated with distinct ChR organization in the two T cell populations. In γδT cells, the overall CCR5/CCR6 profile remained largely preserved despite CD56 expression, whereas in non-γδT cells, CD56 expression was associated with a more distinct CCR5/CCR6 organization, particularly involving the CCR5^+^/CCR6^+^ and CCR5^−^/CCR6^−^ populations. This relative stability of the γδT-cell ChR profile may indicate comparable migration-associated receptor organization between conventional and NK-like γδT cells, although confirmation across a broader range of ChRs will be required. In contrast, the markedly high prevalence of CCR5^+^/CCR6^+^ cells among NK-like non-γδT cells may reflect greater migratory flexibility, potentially enabling trafficking to a wider range of chemokine-producing tissues. Whether this apparent plasticity is unique to the CCR5/CCR6 axis or reflects more widespread changes in ChR organization remains unknown. As discussed above, CD56 expression alone does not demonstrate cytotoxic function, and CCR5/CCR6 expression alone does not establish migratory capacity. Accordingly, future studies combining broader ChR profiling with cytokine production, cytotoxicity markers, and functional chemotaxis assays will be required to determine whether these phenotypic differences translate into distinct migratory or effector capacities.

Across the full analysis, pregnancy-related differences were limited and did not form a consistent pattern across T cell populations, activation states, or CCR5/CCR6 phenotypes. This indicates that the circulating immune phenotypes examined here are broadly maintained during healthy first-trimester pregnancy. This does not argue against the existence of maternal immune adaptation during early pregnancy. Rather, it suggests that the phenotypic parameters examined here are largely unchanged in peripheral blood and therefore may not reflect the principal peripheral markers through which the adaptive processes become apparent. At the same time, the preserved peripheral CCR5/CCR6 organization supports the concept that key immunological adaptations during healthy early pregnancy may be established predominantly within the decidual microenvironment rather than through widespread remodeling of circulating lymphocyte phenotypes.

Several considerations should be acknowledged when interpreting these findings. The cross-sectional design does not allow assessment of longitudinal immune changes, and analysis of peripheral blood alone cannot determine how closely the examined populations reflect decidual immune cells. Furthermore, ChR expression was assessed phenotypically; thus, the observed CCR5/CCR6 profiles characterize trafficking-associated receptor patterns, while their functional relevance to cell migration remains to be confirmed. Finally, PMA/ionomycin provided a strong, standardized, and receptor-independent stimulus that enabled controlled comparison across the investigated lymphocyte subsets but did not reproduce physiological immune activation in vivo. Accordingly, future studies including peri-implantation and later gestational stages, together with paired peripheral blood and decidual samples, should integrate broader ChR profiling with functional analyses of chemotaxis, cytokine production, and cytotoxic effector capacity to clarify the biological relevance of the identified phenotypic patterns.

## 5. Conclusions

Our results demonstrate that γδTCR expression intensity distinguishes circulating γδT-cell subsets with different CCR5/CCR6 co-expression profiles, while their CD4/CD8 and CD56/CD8 phenotypes remain largely comparable. This finding supports the value of broader ChR profiling in γδTCR^dim^ and γδTCR^bright^ cells to determine whether their CCR5/CCR6 differences extend to other migration-related receptors. Functional migration assays will be required to establish whether these receptor-level differences correspond to distinct migratory behavior. Activation-associated differences in CD4/CD8 and CD56-defined γδT-cell phenotypes further indicate that resting and recently activated γδT cells should be considered separately in future phenotypic analyses. Importantly, the highly similar CCR5/CCR6 profiles of conventional and NK-like γδT cells suggest that CD56 expression does not substantially redefine the ChR phenotype in γδT cells. By contrast, the pronounced phenotypic divergence observed in non-γδT cells highlights this conserved organization as a distinguishing feature of γδT cells.

Healthy first-trimester pregnancy did not markedly alter the peripheral immune phenotypes examined, indicating that the investigated circulating ChR and surface-marker patterns are largely maintained during uncomplicated, healthy first-trimester pregnancy, and that major pregnancy-related immune adaptations are likely shaped more strongly at the maternal–fetal interface. Overall, this study provides a phenotypic basis for future work combining extended ChR profiling with functional assessment of migration and effector capacity to define how γδT-cell heterogeneity relates to immune-cell trafficking during healthy pregnancy and, potentially, in pregnancy-related pathological conditions.

## Figures and Tables

**Table 1:** Monoclonal antibodies used in the flow cytometry panel.

Antibody (Target)	Fluorochrome	Isotype	Clone	Manufacturer
anti-human TCRγδ	Brilliant Violet 421	Mouse IgG1	B1	Sony ^1^
anti-human CD45	Alexa Fluor 700	Mouse IgG1	2D1	Sony ^1^
anti-human CD3	PerCP/Cy5.5	Mouse IgG2a	OKT3	Sony ^1^
anti-human CD56	Brilliant Violet 510	Mouse IgG1	HCD56	Sony ^1^
anti-human CCR5	PE	Rat IgG2b	J418F1	Sony ^1^
anti-human CCR6	APC	Mouse IgG2b	G034E3	Sony ^1^
anti-human CD4	PE/Dazzle 594	Mouse IgG1,k	RPA-T4	Sony ^1^
anti-human CD8	APC/Cy7	Mouse IgG1,k	SK1	Sony ^1^
anti-human-CD69	FITC	Mouse IgG1	FN50	Sony ^1^

^1^ Sony Biotechnology Inc., San Jose, CA, USA. FITC (fluorescein isothiocyanate), PE (phycoerythrin), APC (allophycocyanin).

## Data Availability

The original contributions presented in this study are included in the article and its Supplementary Materials. Further inquiries should be directed to the corresponding author.

## Supplementary Materials

The following supporting information can be downloaded at: https://www.mdpi.com/article/10.3390/biology15161386/s1, Supplementary Figure S1. Flow cytometry gating strategies used to identify the analysed cell populations presented in Figure 1, Figure 2 and Figure 3. Supplementary Figure S2. Flow cytometry gating strategies used to identify the analysed cell populations presented in Figure 4 and Figure 5. Supplementary Figure S3. CCR5/CCR6 expression profiles of peripheral blood conventional non-γδT (CD56^−^/γδTCR^−^) and conventional γδT (CD56^−^/γδTCR^+^) cell subsets defined by CD4/CD8 expression in non-pregnant women. Supplementary Figure S4. CCR5/CCR6 Expression profiles of peripheral blood NK-like non-γδT (CD56^+^/γδTCR^−^) and NK-like γδT (CD56^+^/γδTCR^+^) cell subsets defined by CD4/CD8 expression in non-pregnant women.

## Abbreviations

The following abbreviations are used in this manuscript:

CCL	CC Motif Chemokine Ligand
CCR	C-C Motif Chemokine Receptor
CD	Cluster of Differentiation
ChR	Chemokine Receptor
FBS	Fetal Bovine Serum
FMO	Fluorescence Minus One
IFN	Interferon
IL	Interleukin
mAb	monoclonal Antibody
NK	Natural Killer
PBMC	Peripheral Blood Mononuclear Cells
PBS	Phosphate-Buffered Saline
PFA	Paraformaldehyde
PMA	Phorbol Myristate Acetate
TCR	T-Cell Receptor
Tregs	T regulatory cells
PKC	protein kinase C
