# Supplementary material for: CCR5/CCR6 Chemokine Receptor Profiles Differ in γδTCR Intensity-Defined Subsets but Remain Conserved Across Conventional and NK-like γδT Cells During Healthy First-Trimester Pregnancy
Source: Biology (Basel). 2026 Aug 13;15(16):1386. doi: 10.3390/biology15161386 (PMC13509355; doi:10.3390/biology15161386)
Supplement: Supplementary file 1 [file biology-15-01386-s001.zip › biology-4461717-supplementary.pdf]

Supplementary figures

Figure S1

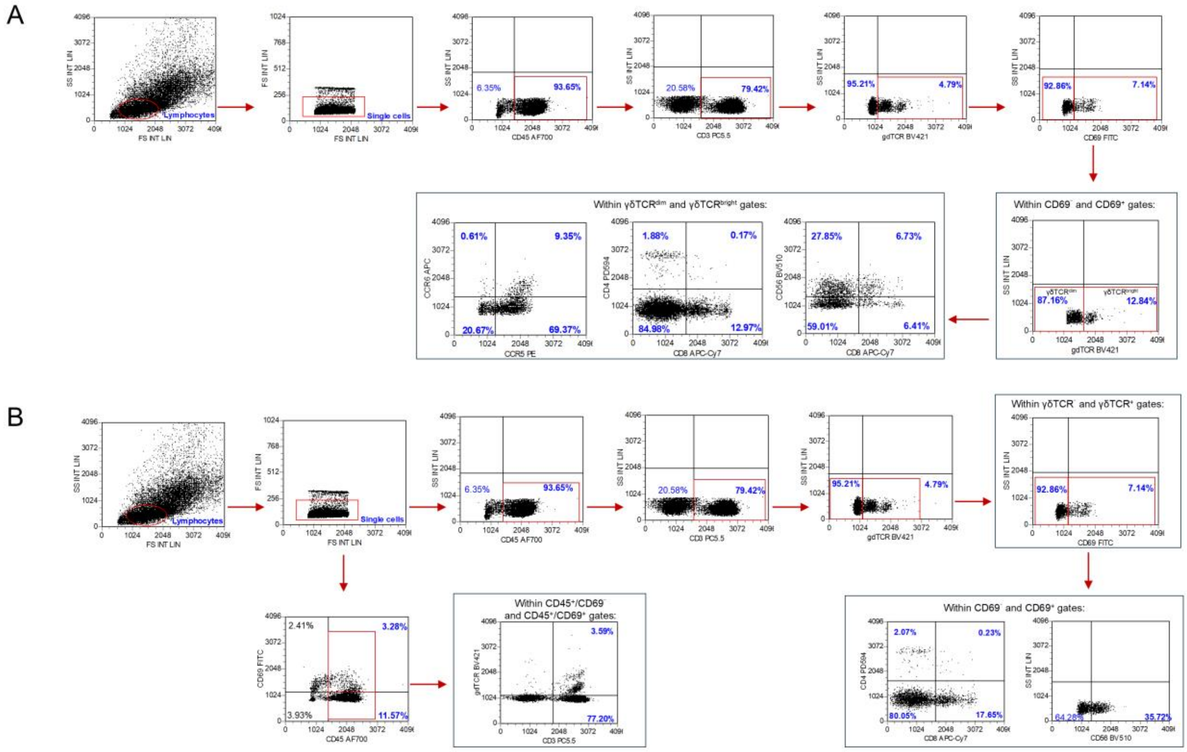

Supplementary Figure S1. Flow cytometry gating strategies used to identify the analysed cell populations presented in Figures 1–3. A) Lymphocytes were initially identified according to forward and side scatter (FSC/SSC) characteristics, followed by the exclusion of doublets through single-cell gating. $CD45^{+}$ leukocytes were subsequently gated, followed by the identification of the $CD3^{+}$ cell population. Within the $CD3^{+}$ population, γδT cells were identified and further subdivided into $CD69^{-}$ (resting) and $CD69^{+}$ (recently activated) subsets. Within both $CD69^{-}$ and $CD69^{+}$ γδT cell subsets, $γδTCR^{dim}$ and $γδTCR^{bright}$ populations were distinguished according to γδTCR expression intensity (gating strategy for Figure 1). The expression of CCR5/CCR6, CD4/CD8, and CD56/CD8 co-expression was subsequently analysed within the $CD69^{-}$ $γδTCR^{dim}$ and $γδTCR^{bright}$ populations (gating strategy for Figure 2). Phenotypic analysis of the $CD69^{+}$ subset was not performed due to the low event count. B) The flow cytometry gating strategy for Panel B followed the same initial steps as those described in Panel A. Further analysis focused on the CD69/CD45-defined cells among single-cell populations, followed by the analysis of CD3/γδTCR co-expression (gating strategy for Figure 3A). Within the $CD3^{+}$ population, $γδTCR^{-}$ cells (non-γδT cells) and $γδTCR^{+}$ cells (γδT cells) were identified. In subsequent analyses, cells were grouped into $CD69^{-}$ and $CD69^{+}$ subsets, followed by characterization of CD56 expression and CD4/CD8 co-expression (gating strategy for Figure 3B–E).

Figure S2

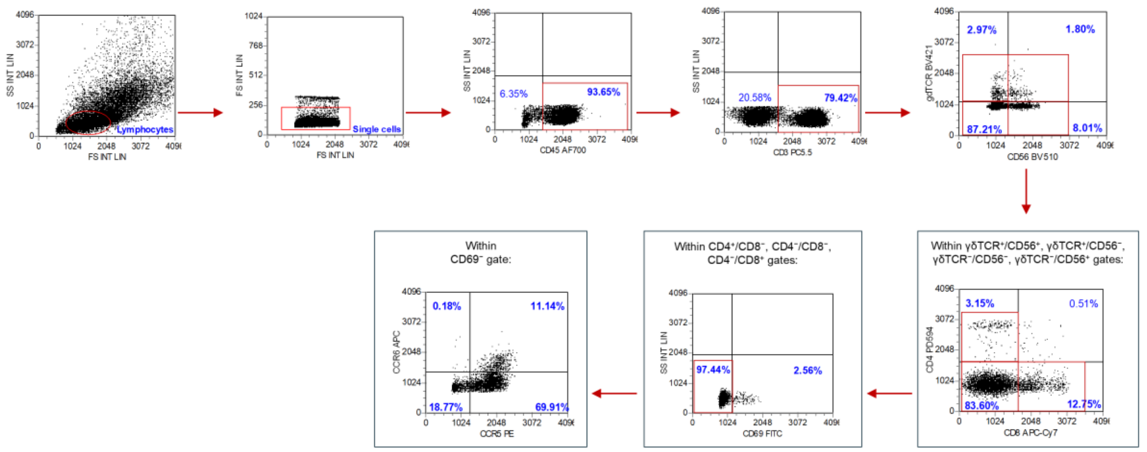

Supplementary Figure S2. Flow cytometry gating strategies used to identify the analysed cell populations presented in Figures 4–5. Lymphocytes were initially identified according to forward and side scatter (FSC/SSC) characteristics, followed by the exclusion of doublets through single-cell gating. $CD45^+$ leukocytes were subsequently gated, followed by the identification of the $CD3^+$ cell population. Within the $CD3^+$ population, four T cell subpopulations were identified according to CD56/γδTCR expression: $CD56^-$/γδTCR^- conventional non-γδT cells and $CD56^-$/γδTCR^+ conventional γδT cells (gating strategy for Figure 4), as well as $CD56^+$/γδTCR^- NK-like non-γδT cells and $CD56^+$/γδTCR^+ NK-like γδT cells (gating strategy for Figure 5). Subsequently, CD4/CD8-defined cell subsets were characterized within each T cell subpopulation, followed by gating on the $CD69^-$ subset. Within this $CD69^-$ gate, CCR5/CCR6 expression profiles were analysed. $CD69^+$ cells were excluded due to low event counts.

Figure S3

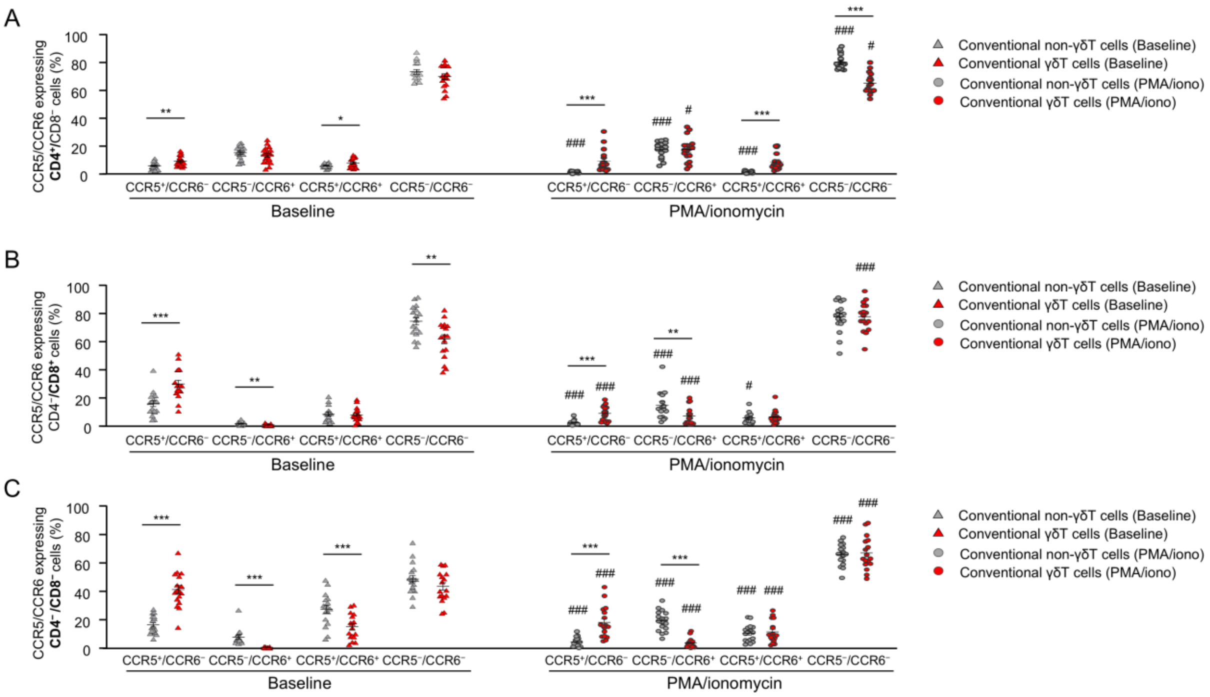

Supplementary Figure S3. CCR5/CCR6 expression profiles of peripheral blood conventional non-γδT (CD56$^-$/γδTCR$^-$) and conventional γδT (CD56$^-$/γδTCR$^+$) cell subsets defined by CD4/CD8 expression in non-pregnant women. Frequencies of CCR5/CCR6-defined cell subsets were assessed within CD4/CD8 populations of conventional non-γδT (CD56$^-$/γδTCR$^-$) and conventional γδT cells (CD56$^-$/γδTCR$^+$) within CD3$^+$ lymphocytes in peripheral blood from non-pregnant women under baseline and PMA/ionomycin-stimulated conditions. CD4$^+$/CD8$^+$ populations were excluded due to insufficient event counts. A) Frequencies of CCR5/CCR6-defined cell subsets within the CD4$^+$/CD8$^-$ population under baseline (CD69$^-$/CD4$^+$/CD8$^-$) and PMA/ionomycin-stimulated conditions (CD69$^+$/CD4$^+$/CD8$^-$). B) Frequencies of CCR5/CCR6-defined cell subsets within the CD4$^-$/CD8$^+$ population under baseline (CD69$^-$/CD4$^-$/CD8$^+$) and PMA/ionomycin-stimulated conditions (CD69$^+$/CD4$^-$/CD8$^+$). C) Frequencies of CCR5/CCR6-defined cell subsets within the CD4$^-$/CD8$^-$ population under baseline (CD69$^-$/CD4$^-$/CD8$^-$) and PMA/ionomycin-stimulated conditions (CD69$^+$/CD4$^-$/CD8$^-$). Data from non-pregnant women (n = 17) are shown as mean ± SEM. The individual data points are represented as triangles (baseline) or circles (PMA/ionomycin stimulated). Normality was assessed using the Shapiro–Wilk test. Statistical significance across multiple phenotypes within each T cell subset was evaluated using one-way ANOVA followed by Bonferroni's post hoc correction. Comparisons between independent subsets and cohorts were performed using the unpaired two-tailed Student's t-test or Mann–Whitney U test, as appropriate. Comparisons between paired groups were performed using the paired Student's t-test for normally distributed data or the Wilcoxon signed-rank test for non-normally distributed data. Statistical significance is indicated as *p ≤ 0.05, **p ≤ 0.01, ***p ≤ 0.001. Hash symbols placed above the PMA/ionomycin-activated data indicate significant differences from baseline within the same T-cell subset and CCR5/CCR6 phenotype ($^{\#}$p ≤ 0.05, $^{\#\#\#}$p ≤ 0.001).

Figure S4

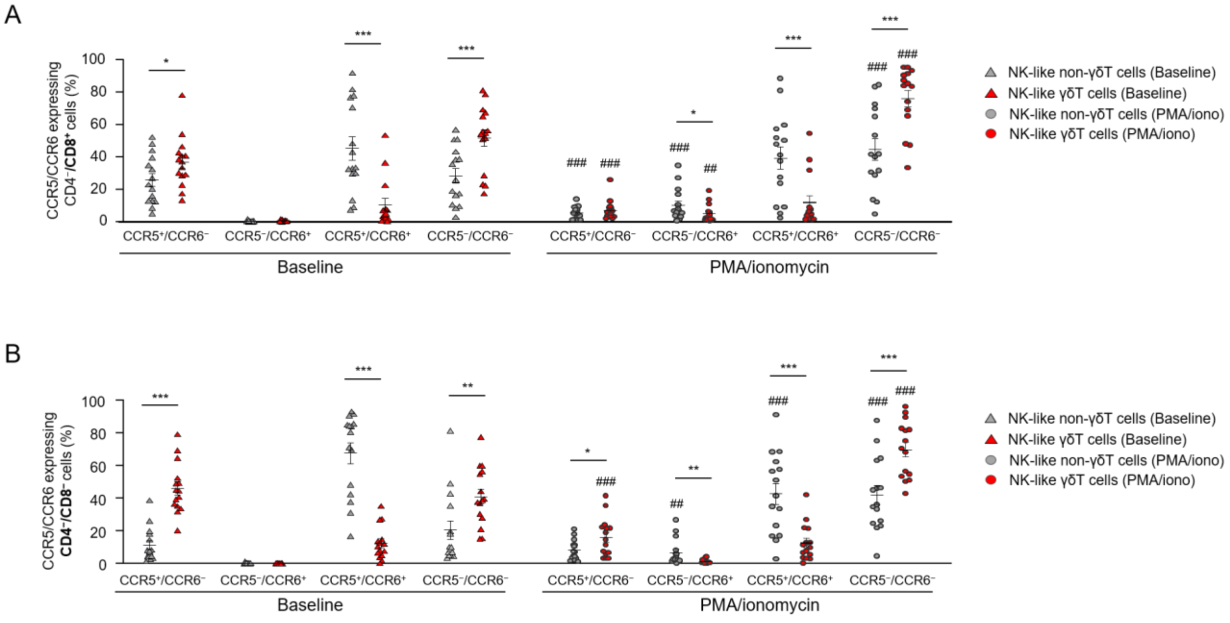

Supplementary Figure S4. CCR5/CCR6 Expression profiles of peripheral blood NK-like γδT (CD56$^+$/γδTCR$^+$) and NK-like non-γδT (CD56$^+$/γδTCR$^-$) cell subsets defined by CD4/CD8 expression in non-pregnant women. Frequencies of CCR5/CCR6-defined cell subsets were assessed within CD4/CD8 populations of NK-like non-γδT (CD56$^-$/γδTCR$^-$) and NK-like γδT cells (CD56$^-$/γδTCR$^+$) within CD3$^+$ lymphocytes in peripheral blood from non-pregnant women under baseline and PMA/ionomycin-stimulated conditions. CD4$^+$/CD8$^-$ and CD4$^+$/CD8$^+$ populations were excluded due to insufficient event counts. A) Frequencies of CCR5/CCR6-defined cell subsets within the CD4$^-$/CD8$^+$ population under baseline (CD69$^-$/CD4$^-$/CD8$^+$) and PMA/ionomycin-stimulated conditions (CD69$^+$/CD4$^-$/CD8$^+$). B) Frequencies of CCR5/CCR6-defined cell subsets within the CD4$^-$/CD8$^-$ population under baseline (CD69$^-$/CD4$^-$/CD8$^-$) and PMA/ionomycin-stimulated conditions (CD69$^+$/CD4$^-$/CD8$^-$). Data from non-pregnant women (n = 15) are shown as mean ± SEM. The individual data points are represented as triangles (baseline) or circles (PMA/ionomycin stimulated). Normality was assessed using the Shapiro–Wilk test. Statistical significance across multiple phenotypes within each T cell subset was evaluated using one-way ANOVA followed by Bonferroni's post hoc correction. Comparisons between independent subsets and cohorts were performed using the unpaired two-tailed Student's t-test or Mann–Whitney U test, as appropriate. Comparisons between paired groups were performed using the paired Student's t-test for normally distributed data or the Wilcoxon signed-rank test for non-normally distributed data. Statistical significance is indicated as *$p \leq 0.05$, **$p \leq 0.01$, ***$p \leq 0.001$. Hash symbols placed above the PMA/ionomycin-activated data indicate significant differences from baseline within the same T-cell subset and CCR5/CCR6 phenotype ($^{\#\#}p \leq 0.01$, $^{\#\#\#}p \leq 0.001$).
